# The Effects of Temperature and Humidity Index on Growth Performance, Colon Microbiota, and Serum Metabolome of Ira Rabbits

**DOI:** 10.3390/ani13121971

**Published:** 2023-06-13

**Authors:** Keyao Li, Mahmoud M. Abdelsattar, Mingming Gu, Wei Zhao, Haoyu Liu, Yafei Li, Pingting Guo, Caiyun Huang, Shaoming Fang, Qianfu Gan

**Affiliations:** 1 College of Animal Science (College of Bee Science), Fujian Agriculture and Forestry University, Fuzhou 350002, China; 3200609004@fafu.edu.cn (K.L.); 13763870270@163.com (M.G.); haoyuliu@163.com (H.L.); 17305027656@163.com (Y.L.); pingtingguo@fafu.edu.cn (P.G.); hcaiyun@yeah.net (C.H.); 15279156575@163.com (S.F.); 2 Department of Animal and Poultry Production, Faculty of Agriculture, South Valley University, Qena 83523, Egypt; m.m.abdelsattar@agr.svu.edu.eg; 3 Key Laboratory of Feed Biotechnology of the Ministry of Agriculture and Rural Affairs, Institute of Feed Research of Chinese Academy of Agricultural Sciences, Beijing 100193, China; zhaowei45@nwafu.edu.cn

**Keywords:** temperature and humidity index, Ira rabbits, colon microbiota, growth performance, serum metabolites

## Abstract

**Simple Summary:**

In this study, multi-omics techniques were used to reveal the effects of actual temperature and humidity index (THI) on intestinal microbe, function and serum metabolism of Ira rabbits. The increase of THI had a significant effect on the intestinal microbial structure of meat rabbits. In response to environmental THI changes, intestinal microorganisms with similar functions were replaced with each other, harmful bacteria were increased, heat-sensitive factors were upregulated, and disease-related biomarkers were increased in serum. In addition, the potential biomarkers of serum metabolism could be predicted with high predictive accuracy. In future production, the rapid detection of biomarkers, which has guiding significance for scientific meat rabbit production, can be attempted to determine whether the use of THI in the small environments of meat rabbits is suitable.

**Abstract:**

This study investigates the effects of different THI values on growth performance, intestinal microbes, and serum metabolism in meat rabbits. The results showed that there were significant differences in THI in different location regions of the rabbit house. The high-THI group (HG) could significantly reduce average daily gain and average daily feed intake in Ira rabbits (*p* < 0.05). The low-THI group (LG) significantly increased the relative abundance of *Blautia* (*p* < 0.05). The HG significantly increased the relative abundance of *Lachnospiraceae NK4A136 group* and reduced bacterial community interaction (*p* < 0.05). The cytokine–cytokine receptor interactions, nuclear factor kappa B signaling pathway, and toll-like receptor signaling pathway in each rabbit’s gut were activated when the THI was 26.14 (*p* < 0.05). Metabolic pathways such as the phenylalanine, tyrosine, and tryptophan biosynthesis and phenylalanine metabolisms were activated when the THI was 27.25 (*p* < 0.05). Meanwhile, the TRPV3 and NGF genes that were associated with heat sensitivity were significantly upregulated (*p* < 0.05). In addition, five metabolites were found to be able to predict THI levels in the environment with an accuracy of 91.7%. In summary, a THI of 26.14 is more suitable for the growth of meat rabbits than a THI of 27.25, providing a reference for the efficient feeding of meat rabbits.

## 1. Introduction

Rabbit meat consumption is widely distributed around the world. According to data from FAOSTAT, in the period 2011–2021, Asian rabbit meat production accounted for 69.9 % of the world’s total [1]. The Ira rabbit is an ideal meat rabbit breed that is valued for its rapid growth rate and high-quality meat [2]. One of the main challenges for the growth of meat rabbit farming is hot environment [3,4]. The prolonged exposure of domestic animals to unusual air temperature and relative humidity values can cause major problems that threaten their comfortable and healthy living and negatively impact their productivity [5]. The primary purpose of the temperature and humidity index (THI) is to measure the changes in environmental conditions [6]. The optimal THI for rabbit growth should be less than 27.8 [7]. When exposed to a THI of 30 or higher, rabbits are no longer able to control their normal body temperature, and develop heat exhaustion [7]. In addition, the artificial simulation environment of an environmental control house cannot accurately reflect the actual environmental conditions in a closed livestock house [8]. The distribution of THI values in the real environment is not uniform [9]. Therefore, the impact of the actual production environment on the health of animals is also worthy of attention.

Rabbits are sensitive to ambient heat because of their lack of sweat glands. Rabbits commonly use increased respiratory rates and vasodilation of the ears as the primary device for thermolysis [3]. As the temperature rises, there is a limit in the heat beyond which a rabbit can be exhausted [10]. The balance of protein, water, energy, and minerals in rabbits is impacted by the thermal environment. In previous studies, heat stress resulted in significant decreases in superoxide dismutase activity, which disturbed the oxidative homeostasis in rabbits [11]. The negative effect of high THI values on the intestine is also obvious. The gut microbiota may account for 10.42% of the difference in Ira rabbits’ weaning weights [12]. Intestinal barrier failure induced by thermal stress may be related to gut microbial imbalance [13]. Chlamydia, Staphylococcus, and Bacteroides are among the bacteria with high THI enrichment that have been identified to be most linked with inflammatory diarrhea [14]. Pathogens in intestinal injuries translocate through tight junction barriers. Animals under thermal stress have activated cytokines, TLR signaling, and heat shock proteins in their intestinal tissues [14]. The average daily gain [15], feed conversion ratio (FCR), and meat quality of a rabbit can all suffer as a result of physiological changes brought on by heat stress [16].

Currently, the knowledge of how the THI affects the health of Ira rabbits remains limited. In this study, the effects of environmental THI on intestinal microorganisms, function, and metabolism in Ira rabbits were studied in a typical closed rabbit house in the southeast coastal area of China. The aim of this paper is to provide ideas and theoretical bases for a judgment index of environmental THI level, which has production guidance significance for scientific meat rabbit breeding.

## 2. Materials and Methods

### 2.1. Animals Feeding

This study was conducted in accordance with, and reviewed by, the Institutional Animal Care and Use Committees at Fujian Agriculture and Forestry University (NO. PZCASFAFU22020, Fuzhou, China).

**Figure 1:**
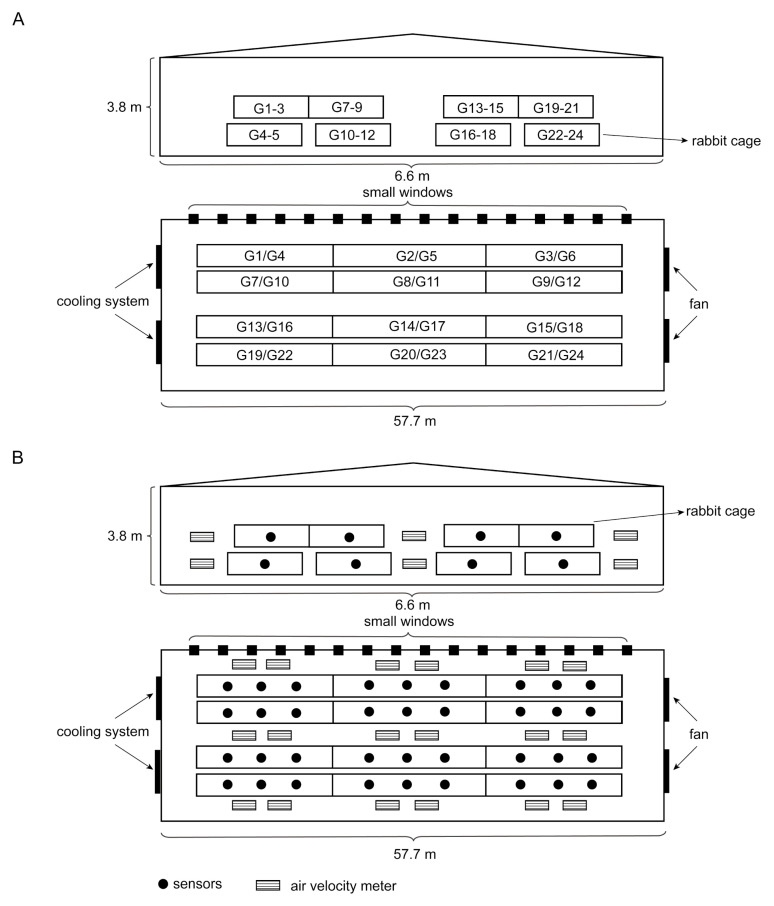
Front view and top view of the rabbit house structure. (**A**) Distribution map of 24 replicate groups. (**B**) Distribution map of environmental factor detectors. G: group.

The present study utilized a total of 648 weaned Ira rabbits (aged 28 days, 0.70 ± 0.072 kg). The rabbits in this experiment were purchased from Fujian Laidewang Animal Husbandry Co., LTD, Sanming, China. The rabbits were randomly divided into 24 replicate groups and housed in the same closed rabbit house (57.7 m long, 6.6 m wide, and 3.8 m high; Figure 1A). All rabbits were reared in 216 cages (the dimensions of the upper cages were 0.5 × 0.55 × 0.35 m and those of the lower cages were 0.5 × 0.9 × 0.4 m, there were 3 animals per cage, and the sexes were randomly distributed). During the experimental period, all the rabbits received freely available water and were fed a fattening diet (Jinjiuwang Feed Co., Ltd., Anqiu, China; Table S1) twice a day. The healthy weaned rabbits had not been given any antibiotics, anticoccidial medications, or prebiotics. This experiment was carried out in Laidewang Animal Husbandry Co., LTD., Sanming, Fujian, China (latitude 25°73′ N; longitude 117°84′ E). 

### 2.2. Data Collection

The rabbit house environment was regulated by an integrated rabbit environmental control system (Sifangxinyu Co., Ltd., Weifang, China). A portable air velocity meter (PCE-VA 20, PCE, Meschede, Germany) was used to measure the house’s temperature (T), relative humidity (RH), and air flow rate every day at intervals of three hours. The amounts of ammonia gas (NH_3_), hydrogen sulfide (H_2_S), and particulate matter were measured using several gas sensors (MultiRAE IR Lite, RAE systems, San Francisco, CA, USA; Figure 1B). Every day from the start to the end, the device recorded concentrations for 12 h (6:00 a.m. to 6:00 p.m.). The temperature and humidity indices of the 24 replicate groups were calculated. A high-THI group (HG) and low-THI group (LG) were formed by choosing the top six duplicate groups with the highest THI values and the bottom six with the lowest THI values. The formula for calculating THI is as follows [17]:

THI = T − [(0.31 − 0.31 × RH) (T − 14.4)]

where THI is the temperature and humidity index, T is the temperature (℃), and RH is the relative humidity (%).

At 28 days of age and 70 days of age, the body weight of each group was recorded. The amounts of food fed to the rabbits and left over were weighed daily. Finally, ADG, ADFI, and feed conversion rate (FCR) were calculated. The growth performance index was calculated by using the following formulae:

ADG = (Average final weight—average weaning weight)/42

ADFI = (Feed quantity—surplus)/42

FCR = ADFI/ADG

where ADG is the average daily gain (kg/d), ADFI is the average daily feed intake (kg/d), and FCR is the feed conversion rate.

### 2.3. Sample Collection

Rabbits were raised to 73 days of age, at which point six rabbits were randomly selected in order to collect samples from each group (LG and HG). In the beginning, experimenters wore long-sleeve experimental suits to avoid being bitten or scratched by rabbits during operation. They would grab the skin of each rabbit‘s neck and take the animal out of the cage, then quickly transfer the rabbit‘s head under their arms to the operating table. Through spinal traction, the rabbit would be placed in a supine position until it became relaxed [18,19]. After the rabbit was prepared well, clear auricular veins were exposed in it, its hair was removed, and alcohol was used to rub and disinfect it. When its veins were filled, 5 mL of blood would be collected in the coagulant collection vessels after vein collection needles were stabbed into the auricular veins against the direction of blood flow. After standing for 1 h, at a blood collection rate 3000 r/min for 10 min, a supernatant would be taken and 3 tubes packed separately [20].Then the rabbit would be killed by the acute blood loss method, its whole abdominal cavity would be opened, and a small colon would be cut. The colon contents would be dipped with a cotton swab, which would be placed in a sterile centrifuge tube. Colon tissue with a length of 1–2 cm would be taken and placed in a sterile centrifuge tube, with 3 samples taken from the rabbit [21]. Prior to experimental analysis, all samples would immediately be submerged in liquid nitrogen. This process was repeated for all six rabbits.

### 2.4. Colon Microbiome Analysis

Using the CTAB/SDS technique, the total genomic DNA of the microorganisms in the colon content samples was extracted. Then, using the barcoded fusion primers 515F (5′-GTGCCAGCMGCCGCGG-3′) and 806R (5′-GGACTACHVGGGTWTCTAAT-3′), the V4 region of the bacterial 16S rDNA gene was amplified. The Phusion^®^ High-Fidelity PCR Master Mix (New England Biolabs Co., LTD, Beijing, China) was used to conduct the PCRs. Then, sequencing libraries were created using the TruSeq^®^ DNA PCR-Free Sample Preparation Kit (Jinao Biotechnology Co., LTD., Wuhan, China), and the NovaSeq6000 (Damai Biotechnology Co., LTD., Shanghai, China) was used to perform the sequencing.

To combine high-quality paired-end readings into tags, FLASH (v.1.2.7) was utilized [22], and QIIME (v.1.9.1) was used to check the quality of these tags. The specific steps of the operation were as follows. (1) Tagging: the first low-quality base site of the original sequence was truncated from a continuous low-quality base number to length 3. (2) Filtering sequence: after the sequences were intercepted, the continuous high-quality sequences with overly small base lengths in the sequence data set (less than 75% of the full length) were filtered out. Finally, each sequence was compared with the species annotation database to detect a chimera sequence, and the chimera sequence was removed to obtain the final high-quality effective sequence.

Using the software UPARSE (v7.0.1001), tags were grouped into operational taxonomic units (OTUs) at 97% sequence identity [23]. OTUs were given a taxonomic categorization using the Mothur technique using the SSUrRNA database of SILVA138 (http://www.arb-silva.de/) accessed on 3 December 2020 [24,25]. Using QIIME (v.1.9.1), the alpha and beta diversity indices were determined. Using the Ape4 software (v.1.7.13), principal coordinates analysis (PCoA) plots were used to assess beta diversity. Using the Vegan package (v.2.5.4), an analysis of similarities (ANOSIM) was carried out based on Bray–Curtis dissimilarity [26]. The R package vegan portrayed the rarefaction curves. At the phylum, class, and genus levels, the relative abundances of bacteria were expressed as percentages. Using Galaxy (http://huttenhower.sph.harvard.edu/galaxy) accessed on 22 June 2022, linear discriminant analysis effect size (LEfSe) analysis was done to identify different bacterial taxa and find species with significantly different abundances [27]. 

The relationships between bacterial taxa were calculated using the SparCC algorithm. A network with edges linking nodes (bacterial taxa) with a Pearson correlation coefficient of over or below 0.8 was drawn using the Igraph package (v.1.2.6). Based on betweenness centrality, as determined by the GirvanNewman algorithm, clusters were created [28].

### 2.5. Colon Transcriptome Analysis

The sequencing of the transcriptome was done on each sample of colon tissue. Utilizing TRIzol^®^ Reagent, total RNA was isolated from the colon (Invitrogen, Waltham, MA, USA). Using a NanoPhotometer^®^ spectrophotometer (Thermo Fisher, Waltham, MA, USA), the total RNA’s purity was evaluated. Using the Bioanalyzer 2100 system’s RNA Nano 6000 Assay Kit (Agilent Technologies, Palo Alto, CA, USA), the integrity and quantity of the total RNA were calculated. The Illumina HiSeq PE150 was used to sequence high-quality libraries. High-quality clean reads were selected from the raw sequences. Then, the HISAT2 software (Version 2.2.0) was used to individually align each clean read to the reference genome in the orientation mode [29]. The approach of fragments per kilobase per million mapped fragments (FPKM) was used to calculate the gene expression level [30].

Using the DESeq2 software (1.32.0), the differential expression analysis of two groups was carried out. To find differentially expressed genes (DEGs) in the LG and HG, the *p* < 0.05 and |log2 > 1| thresholds for substantial variations in gene expression were chosen. KOBAS (http://bioinfo.org/kobas/) was used to carry out the KEGG pathway analysis of the DEGs accessed on 30 July 2022 [31]. When the Bonferroni-corrected *p*-values were less than 0.05, the KEGG pathway enrichment results were considered significant. To determine the relationship between gut bacteria (relative abundance > 0.5%) at the genera level and the host’s DEGs, Pearson correlation analysis was used. The *p*-values, with Bonferroni correction, were less than 0.05, and the correlation coefficient was not fixed. Using the pheatmap package (v.1.0.12), the link between bacteria and genes was demonstrated.

### 2.6. Serum Metabolomics Analysis

100 μL of each serum sample was used to extract metabolites using methanol and 2-chlorobenzalanine after each sample had been thawed in the environment at 4 °C. Around 20% of each metabolite sample was set aside for quality control (QC), and the remaining 80% was used for LC-MS detection [32]. A Thermo Ultimate 3000 system with an ACQUITY UPLC^®^ HSS T3 (150 2.1 mm, 1.8 m, Waters) column kept at 40 °C was used to perform liquid chromatographic separation. The Thermo Q Exactive Plus mass spectrometer was used for mass spectrometry experiments [33]. HCD scans were used in data-dependent acquisition (DDA) MS/MS investigations. Dynamic exclusion was used to exclude some extraneous information from the spectra [33]. 

The Proteowizard program (v3.0.8789) transformed the original data into the mzXML format. For the purpose of peak detection, filtration, and alignment, the R XCMS package (v3.1.3) was utilized [34]. Base peak chromatograms (BPCs) were created by continuously describing the ions in each mass spectrogram with the highest intensity. Metabolite identification (the molecular weight error was = 30 ppm) was done using the Human Metabolome Database (http://www.hmdb.ca, accessed on 16 March 2022), METLIN (http://metlin.scripps.edu, accessed on 16 March 2022), Massbank (http://www.massbank.jp/, accessed on 16 March 2022), LipidMaps (http://www.lipidmaps.org, accessed on 16 March 2022), and mzClound (https://www.mzcloud.org, accessed on 16 March 2022). Using the SIMCA software (v.14.1), orthogonal partial least squares discriminant analysis (OPLS-DA) of the metabolomics data was carried out after standardization via Pareto scaling. The statistical significance was calculated using univariate analysis (*t*-test). Differential metabolites (DMs) between the LG and HG were those with variable importance in projection (VIP) > 1 and *p* < 0.05. Metaboanalyst (www.metaboanalyst.ca, accessed on 16 March 2022) annotated the DMs with KEGG (Kyoto Encyclopedia of Genes and Genomes) pathway analysis. The relationship between gut bacteria and serum DMs was identified using Pearson correlation analysis. Bonferroni-corrected statistics were deemed significant at *p* < 0.05. Using the R random Forest package (v.4.7-1), regression-based random forest models were created to find metabolites that associated with THI.

### 2.7. Statistical Analysis Method

The statistical analysis of ADG was performed using the student’s *t*-test (SPSS V.26.0). Results were presented as the mean standard deviation, and statistical significance was set at *p* < 0.05.

## 3. Results

### 3.1. Group Performance 

There were no significant differences in weaning weight, final weight, and FCR between the LG and HG (*p* > 0.05). The ADG and ADFI of the LG were significantly higher than those of the HG (*p* < 0.05; see Table 1).

### 3.2. Environmental Factors Data Statistics

**Figure 2:**
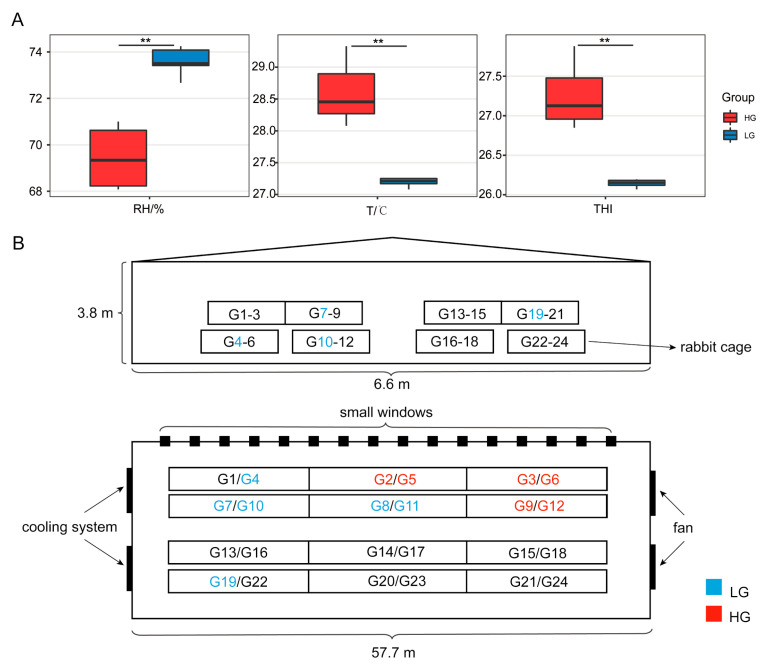
The measurement results of environmental factors in the rabbit house. (**A**) The measurement results of environmental factors. (**B**) The distribution of THI values in the rabbit house. ** *p*.adjust < 0.01.

Between the HG and LG, there were no appreciable variations in NH_3_, H_2_S, PM2.5, and WS (wind speed) (*p* > 0.05, Table S2). However, the temperature, relative humidity, and THI were obviously higher (*p* < 0.001) in the HG than in the LG (Figure 2).

### 3.3. Colon Microbiota Changes

**Figure 3:**
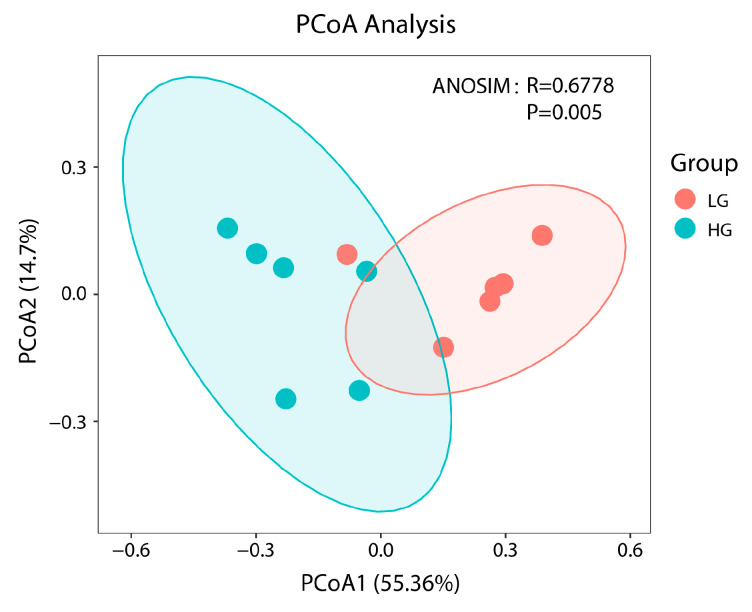
Microbial beta-diversity of rabbits in the low-THI and high-THI environments. PCoA plots are based on Bray–Curtis metrics.

Bacterial clean readings totaled 992,533, with 82,711 being the average number of clean reads (Supplementary Table S3). The rarefaction curve analysis of the sequencing coverage was satisfactory (Supplementary Figure S1). Between the estimations of alpha diversity, there were no differences (*p* > 0.05; Supplementary Table S3). Further PCoA was carried out to confirm the separation of the intestinal bacteria between the LG and HG (Figure 3). The bacterial populations in the rabbits’ guts exhibited a clustering by THI (Bray–Curtis ANOSIM, statistic = 0.6778, *p* = 0.005; Figure 3) which would account for the variation and distinction between two groups.

**Figure 4:**
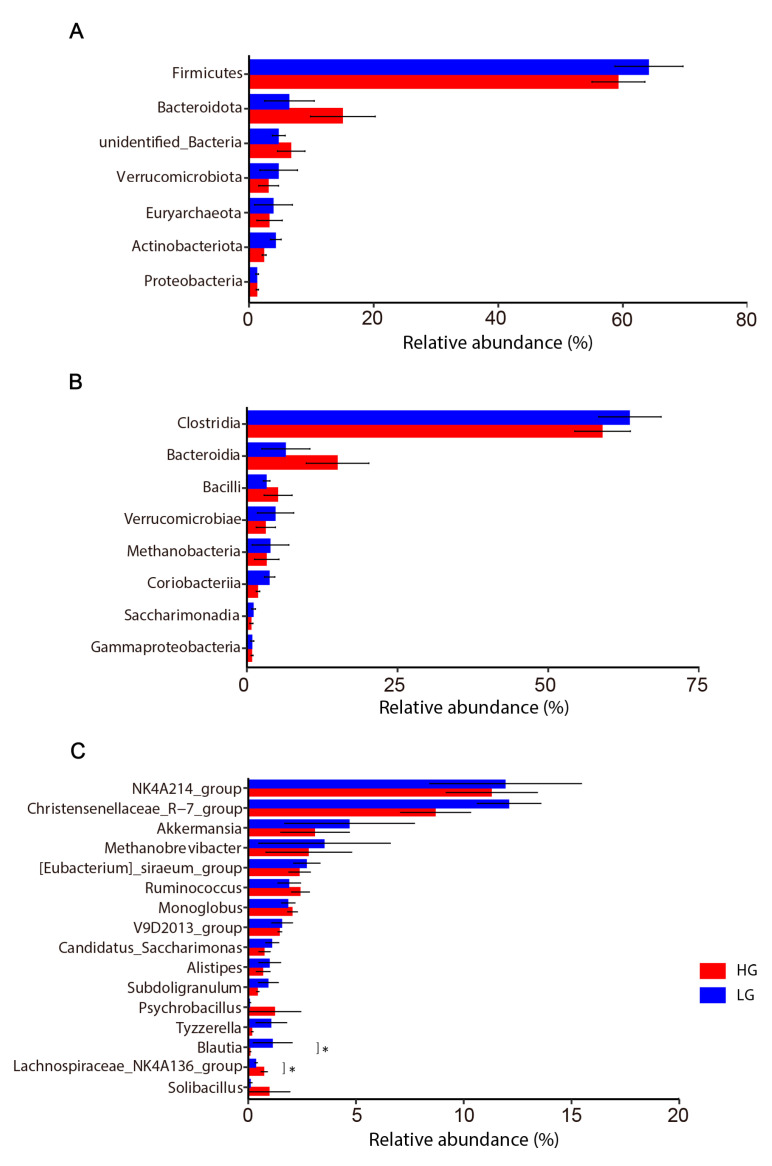
Comparison of intestinal bacterial groups of meat rabbits with different THI values at the (**A**) phylum level, (**B**) class level, and (**C**) genus level. The Wilcoxon rank-sum test was used for comparison. * *p* < 0.05.

39 phyla, 98 classes, and 399 genera of bacteria were found in total, of which 7 phyla, 8 classes, and 16 genera were deemed to be the most prevalent bacterial taxa (relative abundance > 0.5% and prevalence > 20%; see Supplementary Table S4). There were no variations in the relative abundances of these dominant phyla and classes (Figure 4A,B). At the genus level, the abundance of *Lachnospiraceae NK4A136 group* was significantly increased in the HG (*p* < 0.05), while that of *Blautia* was significantly increased in the LG (*p* < 0.05; see Figure 4C).

**Figure 5:**
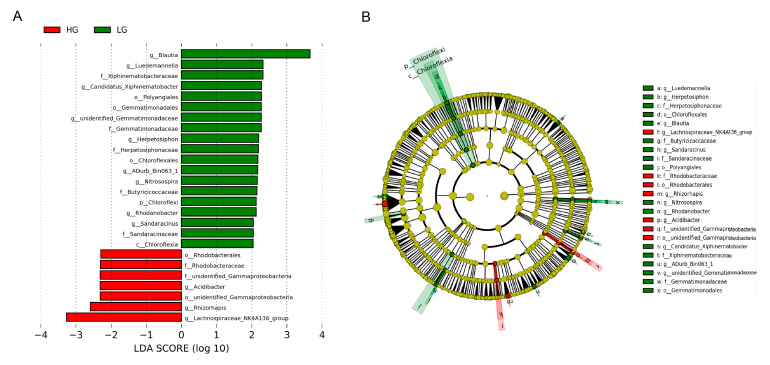
Distinctive gut bacteria composition across different THI groups using LEfSe analysis. |LDA scores| > 2.0. (**A**) Histogram of LDA value distribution by LEfSe analysis; (**B**) Evolutionary cladistics of LEfSe analysis.

LEfSe LDA showed nine and three genera enriched in the LG and HG, respectively. Specifically, Blautia, Luedemannella, Candidatus Xiphinematobacter, unidentified Gemmatimonadaceae, Herpetosiphon, ADurb Bin063 1, Nitrosospira, Rhodanobacter, and Sandaracinus were enriched in the LG, and Acidibacter, Rhizorhapis, and Lachnospiraceae NK4A136 group were enriched in the HG (Figure 5).

**Figure 6:**
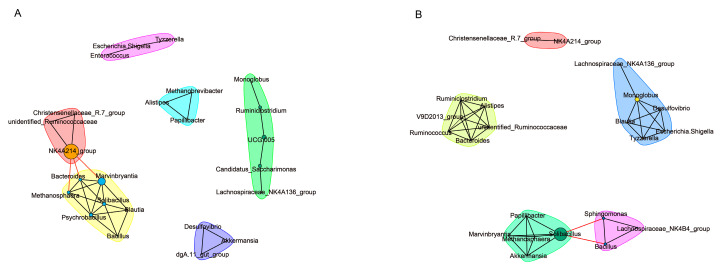
The bacterial interactions in the HG (**A**) and LG groups (**B**). Within the network, each node designates a certain genus, and each line (edge) depicts a substantial coefficiency connection (|Pearson correlation coefficient| > 0.8).

The network analysis of the SparCC method found correlations between genus characteristics (Figure 6). Six and five main subnetworks were detected with THI-associated features in the HG and LG, respectively. Thirty-four connections were found in the HG. The primary subnetwork was created by *NK4A214 group* (pink cluster), *UCG.005*, *Ruminiclostridium*, and *Candidatus Saccharimonas* (green cluster), and *Marvinbryantia*, *Bacteroides*, *Solibacillus*, *Psychrobacillus*, and *Mrthanosphaera* (yellow cluster), which showed strong relationships with other members of the HG community (Figure 6A). Forty-two connections were found in the LG. The three clusters were connected by *Solibacillus* in the light green cluster, *Sphingomonas* and *Bacillus* in the violet cluster, and *Monoglobus* in the light blue cluster (Figure 6B).

**Figure 7:**
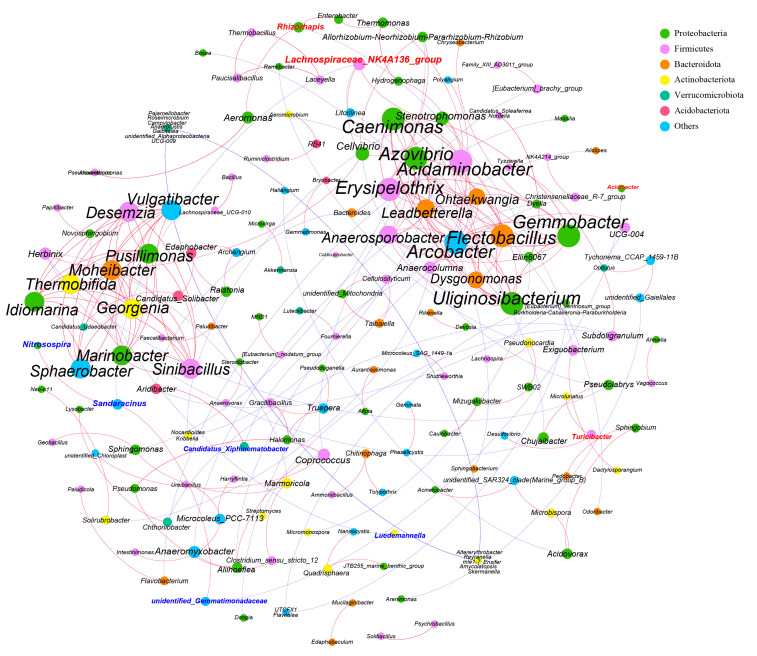
The size of each dot indicates the weight of a genus, and the color of each dot indicates the phylum level to which that flora belongs. The red line describes positive correlation of bacteria, and the blue line describes negative correlation of bacteria (|Pearson correlation coefficient| > 0.8).

A microbial co-occurrence network with the relative abundances of the top 300 genera was constructed (Figure 7). Microbial structure was significantly different across the two models (HG and LG). Genera significantly enriched in the HG (LDA > 2) include *Lachnospiraceae NK4A136 group*, *Acidibacter*, and *Rhizorhapis*. *Candidatus Xiphinematobacter*, *Luedemannella*, *Nitrosospira*, *Sandaracinus* and *unidentity gemmatimonadaceae* were significantly enriched in the LG (LDA > 2). The microbial interaction network consisted of 183 nodes and 534 edges. The ratio of positive correlation to negative correlation in the network was 165:13.

### 3.4. Colon Transcriptome Analysis 

After optimization and quality control, RNA-Seq analysis of the Ira rabbit intestine from the two groups provided 549,499,536 total clean reads and 82,424,930,400 clean bases, totaling 575,438,988 total raw reads and 86,315,848,200 total raw bases. The results of the analysis ranged from 94.12% to 96.33% for clean reads. The mapped rate for the clean reads in the Ira rabbit genome was between 85.07% and 88.65% (Supplementary Table S5).

**Figure 8:**
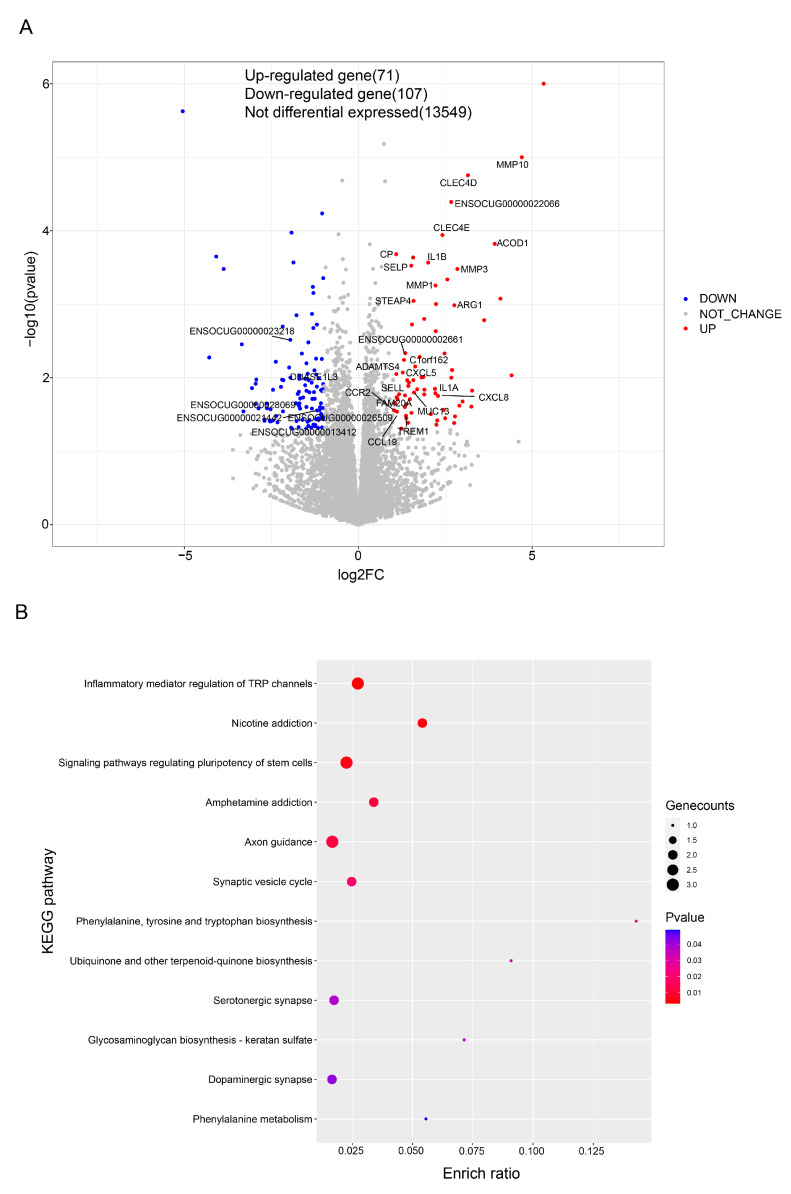

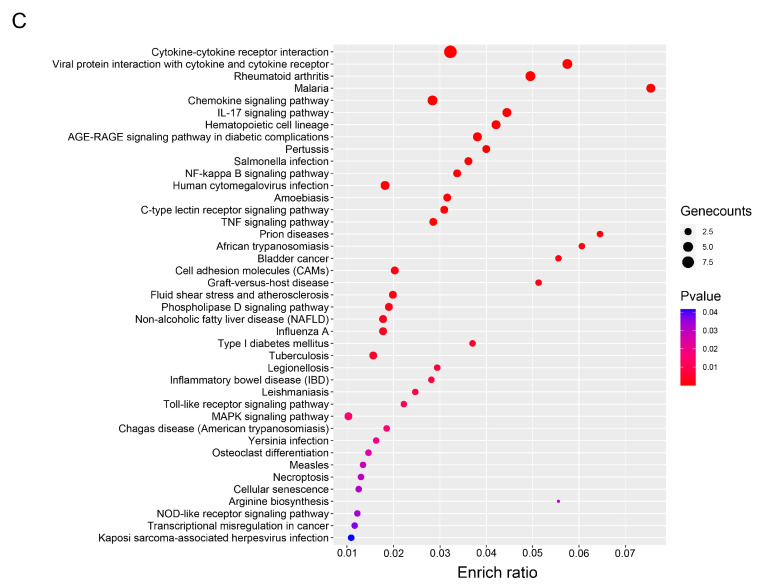
(**A**) DEG volcano maps of the LG and HG groups in the intestines of rabbits. The enriched KEGG pathways of (**B**) upregulated and (**C**) downregulated DEGs.

Between the LG and HG, 178 DEGs were found in the rabbits’ intestines, with 71 upregulated and 107 downregulated genes (Figure 8A). The upregulated and downregulated DEGs were analyzed separately by using KEGG pathway analysis. In the LG, these pathways were designated within five primary categories (Supplementary Table S6) including “Human Diseases” (58.53%), “Organismal Systems” (17.07%), “Environmental Information Processing” (17.07%), “Cellular Processes” (4.89%), and “Metabolism” (2.44%). The most enriched pathways were “Cytokine-cytokine receptor interaction”, “Viral protein interaction with cytokine and cytokine receptor”, “Rheumatoid arthritis”, “Malaria”, and “Chemokine signaling pathway” (Figure 8B).

These KEGG pathways were designated within six primary categories in the LG (Supplementary Table S7), including Organismal Systems (41.67%), Metabolism (33.33%), Human Diseases (16.67%), and Cellular Processes (8.33%). The most enriched pathways were “inflammatory mediator regulation of TRP channels”, “nicotine addiction”, “signaling pathways regulating pluripotency of stem cells”, “amphetamine addiction”, and “axon guidance” (Figure 8C).

### 3.5. Serum Metabolome Analysis 

**Figure 9:**
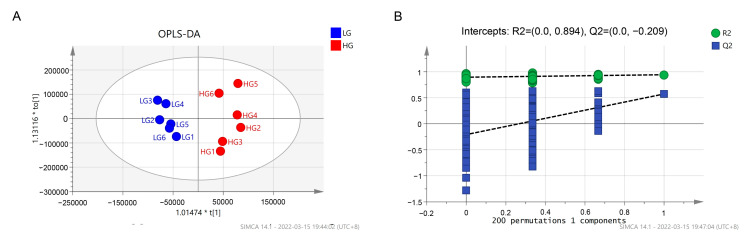
Rabbit serum LC-MS metabolite profiles were used to generate OPLS-DA score plots and accompany permutation testing. (**A**) OPLS-DA score plot. (**B**) Permutation testing.

Metabolomic analysis was used to explore the alterations in serum metabolic profiles between low and high THI values. The serum included a total of 491 metabolites, and differences between the two groups were further demonstrated using OPLS-DA score plots and permutation tests (Figure 9). Ten DMs were identified between the LG and HG with the VIP > 1 combined with *p* < 0.05. Among them, 1-palmitoyl-glycerophosphocholine, N-Alpha-acetyllysine, Acetylphosphate, 16-Hydroxy hexadecanoic acid, and 11-Dehydro-thromboxane B2 were higher in the HG, while Uracil, Kynurenic acid, Inosine, GMP, and beta-Alanine were higher in LG (Table 2). 

**Figure 10:**
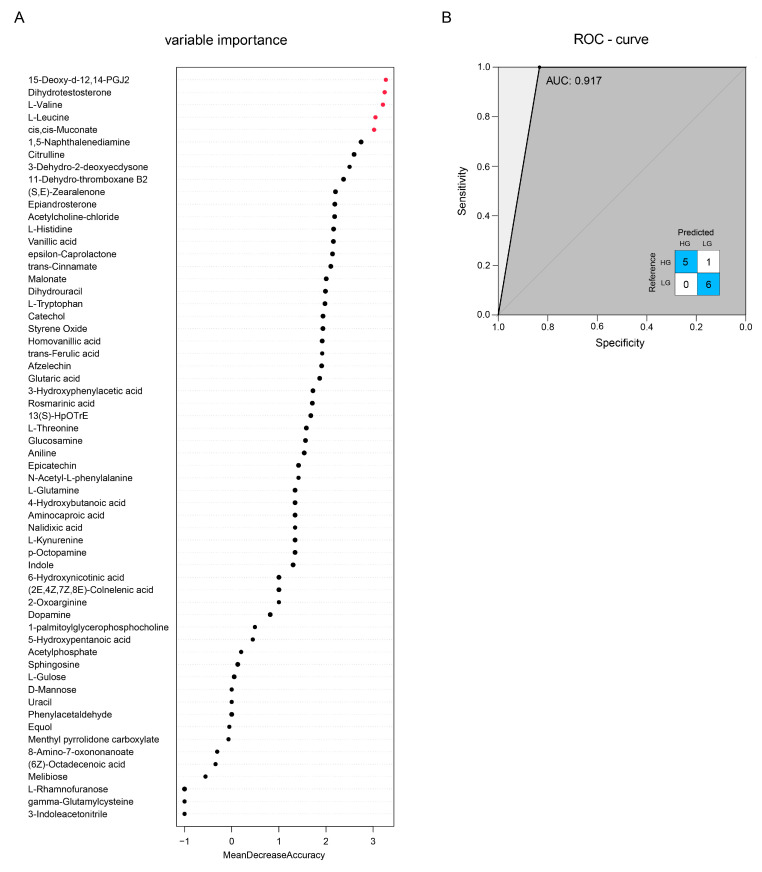
Prediction of key THI-related metabolites based on the random forest model. (**A**) The MDAs of metabolites and five chosen metabolites (shown in red dot) whose MDAs were greater than 3. (**B**) The ROC curve and the confusion matrix.

The THI-associated metabolites were chosen using Spearman correlation analysis from a total of 491 metabolites. Based on Spearman correlation (*p* < 0.05), a total of 60 metabolites were identified as THI-associated metabolites and employed in the random forest model to forecast changes in THI. The mean decrease accuracy (MDA) scores were used to illustrate the importance of metabolites in the model. The random forest model chose five of the THI-associated metabolites with MDAs > 3, including 15-Deoxy-d-12,14-PGJ2, Dihydrotestosterone, L-Valine, L-Leucine, and cis, cis-Muconate (Figure 10A). Together with the inset confusion matrix, the receiver operating characteristic curve [35] exhibited a maximum area under the curve (AUC) of 0.917. The metabolites in five of the six rabbits in the HG were successfully predicted, and the metabolites in all of the LG rabbits were successfully predicted (Figure 10B).

### 3.6. Correlation between the Intestinal Microbiota, DEGs and DMs 

**Figure 11:**
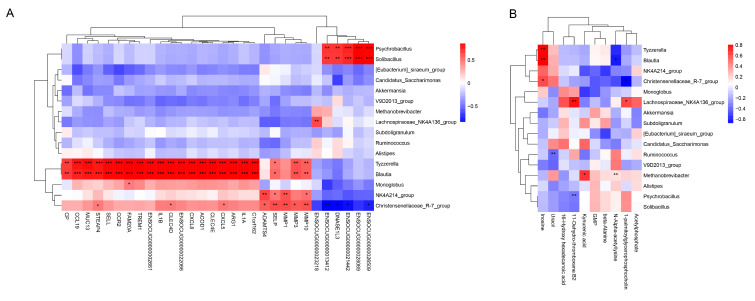
(**A**) Significant correlations between genera and differential expression genes. (**B**) Significant correlations between genera and differential metabolites. Different colors are used to show the correlation coefficients; red indicates a positive relationship and blue indicates a negative relationship. * *p*.adjust < 0.05; ** *p*.adjust < 0.01; *** *p*.adjust < 0.001.

The relationship between gut microbes (relative abundance > 0.5%) and DEGs was revealed by using Pearson correlation analysis. The heatmap showed the top 30 DEGs (Figure 8A) that were significantly different (*p*.adjust < 0.01) from the listed genera. *Psychrobacillus* and *Solibacillu* were positively correlated with DNASE1L3. *Tyzzerella* and *Blautia* were positively correlated with many genes such as MMP10, MMP3, SELP, ADAMTS4, C1orf162, IL1A, ARG1, CXCL5, CLEC4E, ACOD1, CXCL8, CLEC4D, IL1B, TREM1, FAM20A, CCR2, SELL, STEAP4, MUC13, CCL19, and CP. *Monoglobus* was positively correlated with the FAM20A gene; *NK4A214 group* was positively correlated with MMP10, MMP1, SELP, and ADAMTS4. In addition, *Christensenellaceae R-7 group* was positively correlated with MMP3, CXCL5, CLEC4D, and STEAP4, but negatively correlated with DNASE1L3 (Figure 11A).

The relationship between gut microbes (relative abundance > 0.5%) and DMs was revealed by using Pearson correlation analysis. *Tyzzerella* positively correlated with changes in Inosine. *Blautia* positively correlated with inosine but negatively correlated with N-Apha-acetylltsine. *Christensenellaceae R-7 group* positively correlated with inosine but negatively correlated with 1-palmitoyl-glycerophosphocholine. *Lachnospiraceae NK4A136 group* positively correlated with changes in 11-Dehydro-thromboxane B2 and 1-palmitoyl-glycerophosphocholine. *Methanobrevibacter* positively correlated with Kynurenic acid and N-Alpha-acetyllysine. *Ruminococcus* negatively correlated with Uracil. However, *Psychrobacillus* negatively correlated with 11-Dehydro-thromboxane B2 (Figure 11B).

## 4. Discussion

Rabbits are highly sensitive to temperature and relative humidity conditions [3]. The environment within a rabbit house has an enormous impact on rabbit production [5]. This study determined the changes in group performance, colon microbiota, and serum metabolic profiles in Ira rabbits with different THI values.

### 4.1. Group Performance

Daily feed intake (DFI), average daily gain, and feed conversion rate (FCR) are the most important performance parameters used to evaluate the production efficiency of rabbit farms [36]. According to the growth performance of the rabbits in this study, THI 26.14 was more suitable for the growth of meat rabbits than THI 27.25. Inadequate feed intake (FI) caused by heat stress was the main reason for the decreased growth rate. The thermal environment triggered peripheral heat receptors and promoted the secretion of leptin and adiponectin. Leptin stimulated the appetite center of the hypothalamic-pituitary-adrenal axis, and adiponectin regulated eating behavior, resulting in decreased feed intake and ultimately decreased ADG [37]. In addition, extreme reactive oxygen species (ROS) oxidized and destroy cell biomolecules and inhibited ATPase activity in the thermal environment. Finally, intestinal tissue was damaged in the rabbits, and feed utilization efficiency and growth performance were reduced [2]. In addition, the effect of the THI on meat rabbits of different genders was not determined in this study because male and female rabbits had been randomly assigned. Notably, the meat rabbits (28–73 days old) in this experiment had not reached sexual maturity. It is normal for the gender of rabbits to have no effect on production parameters [38], which constitute the physiological moments when the male and female technical indicators begin to differentiate [39].

### 4.2. Colon Microbiota Changes 

Alpha diversity indicators of the microbiota in this investigation did not reveal any appreciable differences (*p* > 0.05); this finding was similar to that of Wen [40]. Changes in physiological parameters do not necessarily lead to changes in specific gut microbiota [41]. In agreement with earlier research, this study discovered that Firmicutes and Bacteroidetes were the most represented intestinal phyla and existed in co-exclusion [42]. Firmicutes are essential for the degradation of dietary fiber and metabolism of lipids during the growth stage in rabbits [43]. Bacteroidetes can increase carbohydrate metabolism and enhance gastrointestinal immunity [44,45]. It has been found that with an increase of THI, the abundance of Bacteroidetes in the rumen of goats significantly increases [41], while the abundance of Bacteroidetes in broilers significantly decreases [46]. In this study, the abundance of Bacteroidetes in meat rabbits increased in the high-THI group, but the change was not significant; this may be due to the differences in physiological characteristics within the species.

Previous studies have shown that THI affects animal gut microbiota composition and function [47]. *Blautia* and *Lachnospiraceae NK4A136 group* were significantly enriched (*p* < 0.05) in the LG and HG, respectively. According to earlier findings, *Blautia* abundance declines as the THI increases. By generating SCFA and increasing intestinal regulatory T cells, *Blautia* plays a critical function in preserving the equilibrium of the gastrointestinal environment and reducing inflammation [48]. Intestinal health has been preserved by the butyrate-producing bacteria of the *Lachnospiraceae NK4A136 group* [49]. One of the primary SCFAs generated by the microbiota, butyrate, is essential for preserving epithelial barrier integrity and inhibiting inflammation [50]. The results of this study revealed that intestinal microbial phylogeny differs among individuals with environmental THI changes. It can be speculated that intestinal microorganisms that are not suited to grow in this THI environment may be replaced by intestinal microorganisms with similar roles in response to environmental THI changes and play a role in protecting intestinal health.

The co-occurrence networks in this study, which suggest potential interactions, revealed different core community structures of genera among rabbits in different THI environments. The high-THI animals had fewer connections than the low-THI animals did (42 vs. 34), indicating that there were fewer microbe–microbe interactions in the former. The core genus of intestinal bacteria in the HG, *NK4A214 group,* was thought to play crucial roles in the fermentation of dietary cellulose and the synthesis of SCFA [12]. *Marvinbryantia* is involved in the conversion of primary bile acids to secondary bile acids and the production of butyric acid [51]. In addition, we found that *Candidatus saccharimonas*, *Ruminiclostridium,* and *Methanosphaera* were core members of the interaction network in the high-THI group. It was found that the bacterial communities of *Candidatus saccharimonas* in the ceca of the meat rabbits increased significantly due to heat stress [52]. *Candidatus saccharimonas* is associated with inflammatory diseases [53]. *Ruminiclostridium* changes are positively correlated with obesity and can aggravate inflammation in mice [54,55]. *Methanosphaera* has been significantly associated with hypercholesterolemia and decreased intestinal trimethylamine-n-oxide (TMAO) concentration [56]. In this study, it was found that high-THI environments affected the colon microbial structure in meat rabbits, and that pathogenic bacteria became the core microflora in the rabbits, possibly inducing intestinal inflammation and other diseases.

### 4.3. Colon Transcriptome Analysis

Using RNA-Seq technology, the impact of THI on the rabbit gut transcriptome was investigated. In this study, IL1A, IL1B, CCR1, CXCL8, CXCR2, and CXCR1 were significantly upregulated in low-THI (26.14) environments. It has been found that immunological response and heat stress are both impacted by the overexpression of CCR1 and IL1B [57]. CXCL8 is a chemokine family member that acts on CXCR1 and CXCR2 receptors. CXCL8 and its receptors contribute to eliminating pathogens and significantly contribute to disease processes and tumorigenesis [58]. This study found a favorable correlation between alterations in intestinal immune-related genes (IL1A, IL1B, and CXCL8) and increasing levels of *Blautia*, indicating that *Blautia* might affect the immunity of rabbits. The aforementioned genes were strongly abundant in pathways like the cytokine–cytokine receptor interaction, NF-κB signaling pathway, and toll-like receptor signaling pathway. During inflammatory and immune reactions to illness, cytokine–cytokine interactions are essential. When cytokine interactions occur, they may have additive, antagonistic, or synergistic effects on physiological processes like eating, body temperature regulation, and sleep [59]. NF-κB activation during recovery from thermal stress is linked to thermotolerance of the NF-κB·IκBα complex, and also with inhibition of ROS accumulation [60]. Antigen detection, dendritic cell maturation, and the beginning of antigen-specific adaptive immune responses are all regulated by toll-like receptors (TLRs). TLR-generated signals are sent through the NF-κB signaling and MAP kinase pathways, which attract co-stimulatory molecules and pro-inflammatory cytokines to sites of inflammation [61]. Thus, the upregulation of these genes and activation of these pathways revealed that THI 26.14 induced immune and inflammatory responses in the rabbit colon. It has been reported that bovine thermal stress first activates HSF1 (heat shock transcription factor 1), then subsequently increases the expression of heat shock proteins, increases glucose and amino acid oxidation, decreases fatty acid metabolism, activates the stress-responsive endocrine system, and finally activates the immune response system [62]. We speculate that immune response pathways are activated in low-THI environments because rabbits are more sensitive to thermal-environmental changes than bovines.

We found that TRPV3 and NGF were significantly upregulated in high-THI (27.25) environments. The TRPV family includes members that encode a dynamic range of thermal sensitivities of sensory neurons. Previous research found that TRPV3-null animals exhibit much less susceptibility to unpleasant temperatures [63]. When there is tissue injury, metabolic stress, and inflammation, NGF is released. NGF enhances the nociceptor response to noxious stimuli, which in turn enhances the experience of pain [64]. Thus, the upregulated expression of TRPV3 and NGF revealed increased sensitivity to environmental THI in rabbit intestines.

We discovered that increased THI also had an impact on the metabolism of phenylalanine and the production of tryptophan, tyrosine, and phenylalanine. The aromatic amino acids (AAA) for protein synthesis include phenylalanine, tyrosine, and tryptophan. Numerous secondary metabolites that are essential for the survival of animals are also produced during the biosynthesis and breakdown of AAA [65]. Phenylalanine, tyrosine, and tryptophan play regulatory roles under heat stress through their co-expression network [66]. Phenylalanine could be metabolized to tyrosine with the help of the enzyme phenylalanine hydroxylase. It has been shown that tyrosine, a chemical that is the precursor of dopamine, norepinephrine, and catecholamine neurotransmitters, can protect against the effects of heat stress [67]. The enhanced metabolism of phenylalanine to tyrosine in bovine mammary epithelial cells (BMECs) was previously reported as a self-defense mechanism against thermal stress [68]. Moreover, the phenylalanine, tyrosine, and tryptophan biosynthesis indole participates in intestinal barrier function and prevents intestinal inflammation [69]. Therefore, we speculate that the induction of tryptophan, tyrosine, and phenylalanine biosynthesis, as well as phenylalanine metabolism, is a self-protective mechanism for the adaptation of the rabbit gut to changes in the THI.

### 4.4. Serum Metabolome Analysis

Serum metabolomics analysis further proved that the THI changes rabbits’ metabolic function. In this study, beta-Alanine, inosine, uracil, and GMP were significantly increased in the LG. Beta-alanine is one of the components of carnosine [70]. The aforementioned compounds have indirect (beta-Alanine) and direct (carnosine) antioxidants, immune boosters, and neurotransmitter actions [71]. Inosine has an anti-inflammatory action and is a member of the class of chemical substances known as purine nucleosides [72]. Elevated levels of beta-alanine and inosine indicate that a low-THI environment affects the immune response in rabbits. The finding in this study of a positive correlation between *Blautia* and inosine suggests that *Blautia* too may affect immunity. In addition, GMP is catalyzed by guanylate cyclase to produce 3′,5′-cyclic guanylate (cGMP). cGMP has critical physiological functions and acts as an intracellular messenger [73]. Uracil, a unique base component of RNA, is a coenzyme for many critical biochemical reactions, such as beta-alanine and pyrimidine metabolism. Uracil is also involved in antioxidant response and the biosynthesis of polysaccharides [74]. According to earlier research, heat stress causes the uracil level in the blood of aquatic animals to drop [75]. In this study, the elevated concentrations of these metabolites suggest that the metabolism of nucleic acids is affected, and the immune function is enhanced, in low-THI environments.

In this study, Acetylphosphate, 1-palmitoylglycerophosphocholine, 11-Dehydro-thromboxane B2, and N-alpha-acetyllysine were significantly increased in the HG. Acetylphosphate (AcP) is a high-energy donor of acetyl and phosphoryl groups that controls the activity of proteins. Studies have demonstrated that AcP can control bacterial pathogenicity by acetylating key transcription factors and decreasing their activity [76]. Besides, environmental variables like pH and temperature seem to have an impact on the condition of the AcP pool [77]. 1-Palmitoylglycerophosphocholine is a cell membrane compound that continuously accumulates during stress responses and contributes to stress tolerance [78]. In this study, a positive correlation was found between 1-palmitoylglycerophosphocholine and *Lachnospiraceae NK4A136 group*. Therefore, it is speculated that 1-palmitoylglycerophosphocholine may contribute to strengthening the intestinal barrier and regulating glucose homeostasis by influencing the abundance of *Lachnospiraceae NK4A136 group*.

N-alpha-acetyllysine is an organic compound classified among the N-acyl-alpha amino acids. N-alpha-acetyllysine may be a biomarker for identifying various nephropathy [79]. In our study, there was a notable negative correlation between *Blautia* and N-apha-acetylltsine, indicating that *Blautia* plays a very important role in host health. 11-Dehydro-thromboxane B2 is a stable metabolite of TXA2, produced in blood and urine, that is used to monitor TXA2 production in vivo [80]. TXA2 is involved in a number of allergy-related illness processes [81]. Therefore, from the elevated levels of these metabolites, it can be inferred that high-THI environments significantly increase the susceptibility of meat rabbits to certain diseases. In addition, changes in intestinal microorganism composition are closely related to host health, and can cause host metabolic dysfunction and increase the risk of disease [82]. Association analysis in this study found that the changes in colon microbes and serum metabolites affected by THI were consistent.

The rapid advancement of machine-learning techniques encourages the use of the metabolome and microbiome to forecast growth and disease risk in various animal species [83]. The interactions between metabolites and environment THI were also investigated in this study using metabolomics. We found that five metabolites could theoretically predict THI adaptation in meat rabbits with an accuracy of 91.7%. These metabolites need to be further verified in more specific production practices.

## 5. Conclusions

In conclusion, THI 26.14 is more suitable for the growth of meat rabbits than THI 27.25. In order to adapt to changes in environmental THI, the stress protection mechanism initiated in meat rabbits is related to the upregulation of immune function and heat-stress-related gene expression, the balance of intestinal microorganisms, and the increasing of some serum metabolites. In future production, markers can be rapidly tested to determine the suitability of specific THIs in the environments of meat rabbits.

## Figures and Tables

**Table 1:** The effects of THI on the growth performance of meat rabbits.

Items	Groups		*p*-Value
	LG	HG	
Weanling weight (kg)	0.73 ± 0.01	0.70 ± 0.01	0.14
Final weight (kg)	2.33 ± 0.10	2.05 ± 0.08	0.09
ADG (g)	46.99 ± 3.10	39.52 ± 2.25	0.01
ADFI (g)	183.34 ± 4.01	151.48 ± 8.37	<0.01
FCR	3.94 ± 0.32	3.87 ± 0.37	0.82

**Table 2:** The differential metabolites identified in the serum metabolomes in Ira rabbits.

Metabolites	HG-Mean	LG-Mean	VIP	*p*-Value
1-palmitoylglycerophosphocholine	5,156,691,973.42	3,276,134,737.27	7.2196	0.0053
N-Alpha-acetyllysine	2,000,058,212.65	1,457,960,754.95	3.9255	0.0494
Acetylphosphate	244,067,959.73	56,877,793.81	1.8856	0.0086
16-Hydroxy hexadecanoic acid	101,453,823.92	70,654,908.76	1.0470	0.0103
11-Dehydro-thromboxane B2	47,712,514.02	15,821,077.55	1.0134	0.0115
Uracil	170,915,960.33	239,164,164.80	1.3382	0.0171
Kynurenic acid	73,581,930.85	195,575,186.82	1.2896	0.0121
Inosine	38,334,884.30	91,956,808.77	1.0915	0.0049
GMP	464,681,530.15	709,621,428.81	1.6793	0.0000
beta-Alanine	644,361,176.34	727,140,676.69	1.6930	0.0053

## Data Availability

Not applicable.

## Supplementary Materials

The following supporting information can be downloaded at: https://www.mdpi.com/article/10.3390/ani13121971/s1, Table S1. The raw material composition and nutritional level of basal diet (Dry matter basis); Table S2. Measured results of environmental factors in rabbit house; Table S3. Summary of 16s rRNA sequencing data in rabbit colon; Table S4. Alpha diversity of colonic microbial of Ira rabbits in two groups; Table S5. Predominant phyla, genera, and species of colon bacteria of Ira rabbit; Table S6. Summary of transcriptomic sequencing data in rabbit colon; Table S7. The enriched KEGG pathway of DEGs in the colon of Ira rabbits in LG group; Table S8. The enriched KEGG pathway of DEGs in the colon of Ira rabbits in HG; Figure S1. Rarefaction curves analysis of colon microbial of Ira rabbits with OTUs of two groups.
