# Supplementary material for: The Effects of Temperature and Humidity Index on Growth Performance, Colon Microbiota, and Serum Metabolome of Ira Rabbits
Source: Animals (Basel). 2023 Jun 13;13(12):1971. doi: 10.3390/ani13121971 (PMC10295653; doi:10.3390/ani13121971)
Supplement: Supplementary file 1 [file animals-13-01971-s001.zip › animals-2361849-supplementary.pdf]

The Effects of Temperature and Humidity Index on Growth Performance, Colon Microbiota, and Serum Metabolome of Ira Rabbits

Keyao Li, Mahmoud M. Abdelsattar, Mingming Gu, Wei Zhao, Haoyu Liu, Yafei Li, Pingting Guo, Caiyun Huang, Shaoming Fang and Qianfu Gan

The journal name: Animal

Supplementary Table S1. The raw material composition and nutritional level of basal diet (Dry matter basis)

Ingredients (%)	Content	Nutrient levels (%)	Content
Corn	12.00	NEmf / (MJ/kg)	10.61
wheat bran	13.90	CP	15.39
Soybean hull	13.00	CF	18.50
Corn germ meal	10.60	EE	2.25
Wheat middlings	6.00	Ca	0.98
Mugwort powder	5.00	P	0.56
Peanut vine powder	20.00	Lysine	0.81
Peanut shell	10.00	Methionine + Cystine	0.61
Cavings	5.00	NDF	36.44
DL-methionine	0.20	ADF	22.09
L-lysine	0.30	ADL	5.60
Premix[1]	4.00		
Total	100.00		

Abbreviations: CP = crude protein; CF = crude fiber; EE = ether extract; NDF = neutral detergent fiber; ADF = acid detergent fiber; ADL = Acid detergent lignin.

[1]The premix provides per kg of diet: Ca 980mg, P 600mg, Fe 70mg, Cu 20mg, Zn 70mg, Mn 10mg, Co 0.15mg, I 0.2mg, Se 0.25mg, VA 10000IU, VD 900IU, VE 50mg, VK2 2mg, thiamine 2mg, riboflavin 6mg, pantothenic acid 50mg, pyridoxine 2mg, VB12 0.02mg, nicotinic acid 50mg, folic acid 44mg, choline 1000mg. Biotin 0.2 mg.

Supplementary Table S2. Measured results of environmental factors in rabbit house.

Items	Group				*P*-value
	LG-Mean	LG-SEM	HG-Mean	HG-SEM	
T, °C	27.20	0.02	28.60	0.16	0.001
RH, %	73.60	0.03	69.44	0.20	0.001
THI	26.14	0.24	27.25	0.55	<0.001
NH_3, ppm	0.00		0.00		
H_2S, ppm	0.00		0.00		
PM2.5, ppm	27.09	0.43	28.24	0.40	0.08
WS, m/s	30.86	0.08	32.50	0.05	0.24

Abbreviations: THI = temperature-humidity index; T = temperature; RH = relative humidity; NH_3 = ammonia gas; H_2S = hydrogen sulfide; PM2.5 = particulate matter; WS = wind speed; LG = low-THI group; HG = High-THI group.

Supplementary Table S3. Summary of 16s rRNA sequencing data in rabbit colon.

Groups	Sample	Raw PE	Raw Tags	Clean Tags	Effective Tags	Base (nt)	AvgLen (nt)	Q20	Q30	GC%	Effective%
LG	LG1	90778	89353	87587	54416	22119911	406	97.8	93.2	55.4	59.9
	LG2	92361	90858	88915	55182	22481226	407	97.9	93.3	55.5	59.8
	LG3	77952	76573	74901	49334	20110722	408	97.7	93.0	54.5	63.3
	LG4	90184	88540	86487	54411	22163712	407	97.7	93.1	54.4	60.3
	LG5	87733	86161	83937	57390	23405671	408	97.7	93.0	54.0	65.4
	LG6	86403	84766	82561	55946	22828163	408	97.7	93.0	53.7	64.8
HG	HG1	87385	86086	84492	55446	22588293	407	97.8	93.3	54.5	63.5
	HG2	89401	87800	85828	52793	21543565	408	97.7	93.1	55.8	59.1
	HG3	80890	79510	77597	51083	20898547	409	97.7	93.1	54.8	63.2
	HG4	77525	76137	74608	48128	19508638	405	97.8	93.2	54.2	62.1
	HG5	93583	91744	89291	57869	23834931	412	97.6	92.9	55.0	61.8
	HG6	79480	78097	76329	50843	21048974	414	97.5	92.6	53.4	64.0

Abbreviations: Raw PE = Original PE reads; Raw Tags = tags sequence spliced; Clean Tags = raw Tags filtered sequence; Effective Tags = follow-up analysis of Tags sequence; Base = the number of bases of Effective Data; AvgLen = average length of Effective Tags; Q20 = error rates of base sequencing less than 1% in Effective Tags; Q30 = error rates of base sequencing less than 0.1% in Effective Tags; GC% = content of GC bases in Effective Tags; Effective % = the number of Effective Tags as a percentage of the number of Raw PE.

Supplementary Table S4. Alpha diversity of colonic microbial of Ira rabbits in two groups.

Index	HG-Mean	HG-SEM	LG-Mean	LG-SEM	*P*-value
ACE	1269.3668	36.6633	1269.8720	29.0112	0.992
Chao1	1239.7997	33.4662	1240.7960	28.9157	0.982
Shannon	6.6430	0.2301	6.5313	0.2278	0.737
Simpson	0.9533	0.0110	0.9522	0.0124	0.945
Coverage, %	99.48	0.3070	99.48	0.3070	1.0000

Supplementary Table S5. Predominant phyla, genera, and species of colon bacteria of Ira rabbit.

Taxa name	HG-Mean	HG-SEM	LG-Mean	LG-SEM	*P*-value
Phyla (%)					
Firmicutes	59.2853	4.2470	64.1804	5.4509	0.3367
Bacteroidota	15.0627	5.1886	6.4751	3.9859	0.1093
unidentified_Bacteria	6.7739	2.1896	4.7749	1.0246	0.6310
Verrucomicrobiota	3.1465	1.5889	4.7617	3.0157	0.8728
Euryarchaeota	3.2931	2.0291	3.9245	3.0395	1.0000
Actinobacteriota	2.4296	0.3089	4.3008	0.8642	0.3905
Proteobacteria	1.3276	0.1936	1.3040	0.2192	0.8726
Class (%)					
Clostridia	59.0105	4.6097	63.5417	5.1889	0.4848
Bacteroidia	15.0611	5.1898	6.4735	3.9852	0.1320
Bacilli	5.1874	2.3208	3.2911	0.5448	0.9372
Verrucomicrobiae	3.1465	1.5887	4.7617	3.0148	0.9372
Methanobacteria	3.2931	2.0295	3.9245	3.0405	1.0000
Coriobacteriia	1.8508	0.2970	3.7835	0.8494	0.1320
Saccharimonadia	0.7593	0.2608	1.1151	0.3021	0.3095
Gammaproteobacteria	0.8679	0.1690	0.9086	0.2421	0.9372
Genera (%)					
NK4A214_group	11.9405	3.5203	11.3011	2.1169	0.8182
Christensenellaceae_R-7_group	12.1055	1.4734	8.6950	1.6264	0.3095
Akkermansia	4.7010	3.0096	3.0986	1.5921	0.9372
Methanobrevibacter	3.5419	3.0513	2.8070	1.9937	0.6991
[Eubacterium]_siraeum_group	2.7183	0.6079	2.3824	0.4978	0.5887
Ruminococcus	1.9024	0.5239	2.4232	0.4128	0.4848
Monoglobus	1.8584	0.3106	2.0549	0.2274	0.4848
V9D2013_group	1.5728	0.4828	1.4650	0.0851	0.6884
Candidatus_Saccharimonas	1.1107	0.3024	0.7565	0.2611	0.3095
Alistipes	0.9973	0.5045	0.6926	0.3177	0.6991
Subdoligranulum	0.9350	0.4619	0.4469	0.0502	0.3939
Psychrobacillus	0.0815	0.0312	1.2433	1.1934	0.8726
Tyzzerella	1.0712	0.7120	0.1837	0.0399	0.1735
Blautia	1.1363	0.8974	0.1010	0.0181	0.0247
Lachnospiraceae_NK4A136_group	0.3659	0.0527	0.7397	0.1419	0.0411
Solibacillus	0.1150	0.0573	0.9849	0.9454	0.5725

Supplementary Table S6. Summary of transcriptomic sequencing data in Ira rabbits.

Group	Sample	Raw reads	Raw bases	Clean reads	Clean bases	Clean Reads Rate (%)	Mapped reads	Mapped rate (%)
LG	LG1	48,170,126	7,225,518,900	45,734,072	6,860,110,800	94.94	40,173,963	87.84
	LG2	48,807,314	7,321,097,100	47,016,960	7,052,544,000	96.33	41,678,909	88.65
	LG3	50,036,826	7,505,523,900	47,955,762	7,193,364,300	95.84	42,117,698	87.83
	LG4	49,688,934	7,453,340,100	47,383,622	7,107,543,300	95.36	38,600,816	87.60
	LG5	50,023,704	7,503,555,600	47,635,636	7,145,345,400	95.23	41,501,982	87.12
	LG6	47,448,214	7,117,232,100	45,677,288	6,851,593,200	96.27	39,933,445	87.43
HG	HG1	38,388,506	5,758,275,900	36,405,932	5,460,889,800	94.84	31,808,335	87.37
	HG2	50,348,740	7,552,311,000	47,875,524	7,181,328,600	95.09	41,988,060	87.70
	HG3	50,644,052	7,596,607,800	47,665,714	7,149,857,100	94.12	41,913,613	87.93
	HG4	46,938,052	7,040,707,800	45,162,232	6,774,334,800	96.22	38,418,735	85.07
	HG5	48,536,028	7,280,404,200	46,681,912	7,002,286,800	96.18	40,865,958	87.54
	HG6	46,408,492	6,961,273,800	44,304,882	6,645,732,300	95.47	39,253,050	88.60

Supplementary Table S7. The enriched KEGG pathway of DEGs in the colon of Ira rabbits in LG.

Description	ID	Category		*P*-value
		Level-1	Level-2	
Cytokine-cytokine receptor interaction	ocu04060	Environmental Information Processing	Signaling molecules and interaction	0.0000
Viral protein interaction with cytokine and cytokine receptor	ocu04061	Environmental Information Processing	Signaling molecules and interaction	0.0000
Rheumatoid arthritis	ocu05323	Human Diseases	Immune disease	0.0000
Malaria	ocu05144	Human Diseases	Infectious disease: parasitic	0.0000
Chemokine signaling pathway	ocu04062	Organismal Systems	Immune system	0.0000
IL-17 signaling pathway	ocu04657	Organismal Systems	Immune system	0.0000
Hematopoietic cell lineage	ocu04640	Organismal Systems	Immune system	0.0000
AGE-RAGE signaling pathway in diabetic complications	ocu04933	Human Diseases	Endocrine and metabolic disease	0.0000
Pertussis	ocu05133	Human Diseases	Infectious disease: bacterial	0.0003
Salmonella infection	ocu05132	Human Diseases	Infectious disease: bacterial	0.0005
NF-kappa B signaling pathway	ocu04064	Environmental Information Processing	Signal transduction	0.0006
Human cytomegalovirus infection	ocu05163	Human Diseases	Infectious disease: viral	0.0006
Amoebiasis	ocu05146	Human Diseases	Infectious disease: parasitic	0.0007
C-type lectin receptor signaling pathway	ocu04625	Organismal Systems	Immune system	0.0007

TNF signaling pathway	ocu04668	Environmental Information Processing	Signal transduction	0.0009
Prion diseases	ocu05020	Human Diseases	Neurodegenerative disease	0.0015
African trypanosomiasis	ocu05143	Human Diseases	Infectious disease: parasitic	0.0017
Bladder cancer	ocu05219	Human Diseases	Cancer: specific types	0.0020
Cell adhesion molecules (CAMs)	ocu04514	Environmental Information Processing	Signaling molecules and interaction	0.0023
Graft-versus-host disease	ocu05332	Human Diseases	Immune disease	0.0023
Fluid shear stress and atherosclerosis	ocu05418	Human Diseases	Cardiovascular disease	0.0024
Phospholipase D signaling pathway	ocu04072	Environmental Information Processing	Signal transduction	0.0028
Non-alcoholic fatty liver disease (NAFLD)	ocu04932	Human Diseases	Endocrine and metabolic disease	0.0033
Influenza A	ocu05164	Human Diseases	Infectious disease: viral	0.0033
Type I diabetes mellitus	ocu04940	Human Diseases	Endocrine and metabolic disease	0.0043
Tuberculosis	ocu05152	Human Diseases	Infectious disease: bacterial	0.0047
Legionellosis	ocu05134	Human Diseases	Infectious disease: bacterial	0.0066
Inflammatory bowel disease (IBD)	ocu05321	Human Diseases	Immune disease	0.0072
Leishmaniasis	ocu05140	Human Diseases	Infectious disease: parasitic	0.0092
Toll-like receptor signaling pathway	ocu04620	Organismal Systems	Immune system	0.0112
MAPK signaling pathway	ocu04010	Environmental Information Processing	Signal transduction	0.0146

Chagas disease (American trypanosomiasis)	ocu05142	Human Diseases	Infectious disease: parasitic	0.0157
Yersinia infection	ocu05135	Human Diseases	Infectious disease: bacterial	0.0200
Osteoclast differentiation	ocu04380	Organismal Systems	Development and regeneration	0.0244
Measles	ocu05162	Human Diseases	Infectious disease: viral	0.0285
Necroptosis	ocu04217	Cellular Processes	Cell growth and death	0.0302
Cellular senescence	ocu04218	Cellular Processes	Cell growth and death	0.0324
Arginine biosynthesis	ocu00220	Metabolism	Amino acid metabolism	0.0326
NOD-like receptor signaling pathway	ocu04621	Organismal Systems	Immune system	0.0338
Transcriptional misregulation in cancer	ocu05202	Human Diseases	Cancer: overview	0.0369
Kaposi sarcoma-associated herpesvirus infection	ocu05167	Human Diseases	Infectious disease: viral	0.0416

Abbreviations: Level-1 = KEGG level-1 pathway; Level-2 = KEGG level-2 pathway.

Supplementary Table S8. The enriched KEGG pathway of DEGs in the colon of Ira rabbits in HG.

Description	ID	Category		*P*-value
		Level-1	Level-2	
Inflammatory mediator regulation of TRP channels	ocu04750	Organismal Systems	Sensory system	0.0033
Nicotine addiction	ocu05033	Human Diseases	Substance dependence	0.0047
Signaling pathways regulating pluripotency of stem cells	ocu04550	Cellular Processes	Cellular community - eukaryotes	0.0055
Amphetamine addiction	ocu05031	Human Diseases	Substance dependence	0.0113
Axon guidance	ocu04360	Organismal Systems	Development and regeneration	0.0125
Synaptic vesicle cycle	ocu04721	Organismal Systems	Nervous system	0.0202
Phenylalanine, tyrosine and tryptophan biosynthesis	ocu00400	Metabolism	Amino acid metabolism	0.0209
Ubiquinone and other terpenoid-quinone biosynthesis	ocu00130	Metabolism	Metabolism of cofactors and vitamins	0.0311
Serotonergic synapse	ocu04726	Organismal Systems	Nervous system	0.0381
Glycosaminoglycan biosynthesis - keratan sulfate	ocu00533	Metabolism	Glycan biosynthesis and metabolism	0.0388
Dopaminergic synapse	ocu04728	Organismal Systems	Nervous system	0.0417
Phenylalanine metabolism	ocu00360	Metabolism	Amino acid metabolism	0.0488

Abbreviations: Level-1 = KEGG level-1 pathway; Level-2 = KEGG level-2 pathway.

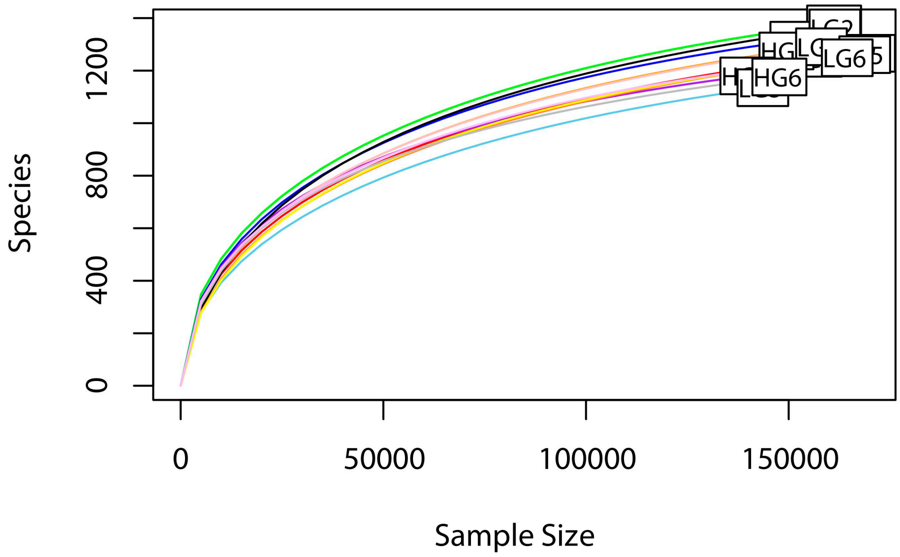

Supplementary Figure S1. Rarefaction curves analysis of colon microbial of Ira rabbits with OTUs of two groups.
